# Supplementary material for: The rodent object-in-context task: A systematic review and meta-analysis of important variables
Source: PLoS One. 2021 Jul 16;16(7):e0249102. doi: 10.1371/journal.pone.0249102 (PMC8284613; doi:10.1371/journal.pone.0249102)
Supplement: S1 File — (PDF) [file pone.0249102.s002.pdf]

Supporting Information (appendix):

Sep & Vellinga, Sarabdjitsingh, Joëls (2021). *The rodent object-in-context task: a systematic review and meta-analysis of important variables.* PLOS ONE.

Table of Contents

A1. Complete PubMed Search string 2

A2. Data-extraction codebook 2

A3. DR conversion formula's 5

A4. Summary quality scores formulas 6

A5. Summary score formulas 6

A6. Study characteristics 6

A7. Systematic review table 10

A8. Study quality 15

A9. Random forest model convergence plot 15

A10. PRISMA 2009 Checklist 16

A1. Complete PubMed Search string

(
("Memory, Episodic"[MeSH] OR "Memory, Long-Term"[MeSH] OR "Memory, Short-Term"[MeSH] OR "Spatial Memory"[MeSH] OR "Discrimination Learning"[MeSH] OR "Spatial Learning"[MeSH] OR "Episodic memory"[all Fields] OR "Memories"[all fields] OR "Episodic like memory"[all Fields] OR "long term memory"[all Fields] OR "short term memory"[all Fields] OR "spatial memory"[all Fields] OR "working memory"[all Fields] OR "discrimination learning"[all Fields] OR "spatial learning"[all Fields] OR "memory"[all Fields] OR "recognition memory"[all Fields] OR "Association learning"[MeSH] OR "Discrimination Learning"[MeSH] OR "Learning"[MeSH] OR "Learn"[all fields] OR "Learning"[all fields] OR "Learning task"[all Fields] OR "association learning"[all Fields] OR "discrimination learning"[all Fields] OR "discrimination task"[all fields])
AND
("rodentia"[MeSH] OR mice[all fields] OR mus[all fields] OR mouse[all fields] OR murine[all fields] OR woodmouse[all fields] OR rats[all fields] OR rat[all fields] OR murinae[all fields] OR muridae[all fields] OR cottonrat[all fields] OR cottonrats[all fields] OR hamster[all fields] OR hamsters[all fields] OR cricetinae[all fields] OR rodentia[all fields] OR rodent[all fields] OR rodents[all fields])
AND
(
"OIC"[all fields] OR "object in context"[all fields] "object context"[all fields] OR "object place context"[all fields] OR "object background"[all fields] OR "context mismatch"[all fields] OR "object-in-context"[all fields] OR "context item"[all fields] OR "context recognition"[all fields]
OR
(("object"[all fields] OR "item"[all fields]) AND "context"[all fields] AND ("task"[all fields] OR "test"[all fields] OR "recognition"[all fields] OR "discrimination"[all fields]))
)
)
NOT ("meta-analys*"[ti] OR "review"[ti] OR Review [Publication Type] OR systematic[sb] OR "meta-analysis"[Publication Type])

A2. Data-extraction codebook

Table A1. Data-extraction codebook
** Empty cells indicate that information was not (re)coded but extracted as described in the article.*
# Variables with more than 1/3 missing values that were not included in the random forest analysis.

Variable	General explanation	Coding*
Publication details		
Reference	APA reference (copied from google scholar)	
PMID	Pubmed ID	
Authors	From Pubmed	
Affiliation	From Pubmed	
year	Of publication	
journal	From Pubmed	
EXP_group	Identifier of each unique animal group in a study (unique in combination with PMID).	
Animal factors		
Species	Animal species. Note, although all rodent species were eligible, only rat and mice papers were present in the dataset.	Rat / Mice
Strain	Strain as reported in publication	
Sex	Sex of animals	M (Male) / F (Female) / Both
Origin	Origin of the breeding animals	B (in-house breeding) / P (purchased animals)
Age	Grouped age	Juvenile (0-21 days); Adolescent (22-56 days); Adult (57-365 days); Senior (>365 days).

Prev.Exp	Participation in previous experiments other than object recognition? (see comment for type of experiment)	Yes / No
Prev.OR	previous participation in object recognition experiments	Yes / No
Type.Control	Type of control group	U (Untouched) / SO (sham operated) / SI (saline injected)
<u>Experimental factors</u>		
AdLib	Ad libitum access to food or water?	Yes / No
Housing	Housing prior to experiment	G (group-housed) / S (single-housed)
RevRhythm	Reversed day-night rhythm?	Yes / No
nC	Sample size of control group; Note,the mean size is calculated if only a range is provided.	
<u>Task-related experimental factors</u>		
Context.Floor	Experimental box; contextual differences; floors box A/B?	Different / Similar
Context.Wall	Experimental box; contextual differences walls box A/B?	Different / Similar
Context.Room	Experimental box; contextual differences; rooms box A/B?	Different / Similar
Context.Size.A	Floor size of box A	Size (cm2)
Context.Size.B	Floor size of box B	Size (cm2)
Context.Shape	Experimental box; shape difference between box A/B?	Different / Similar
Object.Material	Objects; characteristics; different materials?	Different / Similar
Object.Size #	Objects; characteristics; different sizes?	Different / Similar
Object.Position #	Objects; Position objects relative to room / each other	
Animal.position #	Position how the animal is placed in the box.	
Handling #	Handling prior experiments	Yes / No
Testroom.Habituation #	Habituation to test room	Yes / No
Transport.Habituation #	Habituation to transportation	Yes / No
Context.Habituation.Freq	Habituation to experimental boxes; Number of visits per box	
Habituation.Time.Trial	Habituation to experimental boxes; time per trial	Time (min)
Habituation.Time.Total.Context	Total habituation time per box	Time (min)
Habituation.with.Cagemates	Habituation with cage mates?	Yes / No / Partly
Object.Habituation	Habituation to experimental boxes; objects present?	Yes / No / Partly
Learning.Time.Trial	Learning (acquisition) phase per trial	Time (min)
Learning.Learning.Delay	time between learning trials	Time (min)
Learning.Order.Counterbalanced	Learning sequence counterbalanced?	Yes / No
Learning.Exploration.Time.A #	Learning phase; average exploration time box A	Time (sec)
Learning.Exploration.Time.B #	Learning phase; average exploration time box B	Time (sec)

Learning.Exploration.Time.Total #	Sum of average exploration time in box A and B	Time (sec)
Learning.Memory.Delay	Retention time between last learning trial and the memory trial	Time (min)
Memory.Time.Trial	Memory phase; time of trial	Time (min)
Memory.Exploration.Time.A #	Memory trial; exploration time box A	Time (sec)
Memory.Exploration.Time.B #	Memory trial; exploration time box B	Time (sec)
Memory.Exploration.Time.Familiar #	Memory trial; exploration time in context object	Time (sec)
Memory.Exploration.Time.New #	Memory trial; exploration time out of context object	Time (sec)
Memory.Exploration.Time.Total #	Total exploration time in memory trial (average of expl box A and B or sum in and out of context objects)	Time (sec)
Context.Order	Context order: AA (Last box learning similar to memory box), AB (Last box learning different from memory box), Both (randomized for context order). Note, the counterbalanced use of AB and BA is coded as AB; Both us used for AA and AB combinations.]	AA/AB/Both
Scoring	Behavioral Scoring	Manual/Digital/Both
Program	Behavioral scoring, if digital: Name of scoring program + version	
Observers #	Behavioral scoring, if manual: Number of observers	1; >1
Def_Expl	Definition of exploration	
SmellCtrl	“Smell control”: cleaning box / object or changing bedding)	Yes / No
ReuseBedding #	Reuse of OIC bedding?	Yes / No (if yes, number of trials added)
Scoring		
DR.Formula	Formula for DI/DR	
Page_outcome	Location (Page number) of outcome (DR) in article	
Memory.Type	Memory domain, based on retention time between learning and memory phase: Short-term memory (STM), ‘medium, long-term memory (LTM)	STM (0-10min delay) / ‘medium’ (10 min. -1hr delay) / LTM (>1hr delay)
DR	Discrimination ratio, as reported in article or extracted from figures with PlotDigitizer	
SEM	Standard error of the mean from DR	
DR_B	(Converted) DR_B_n/(n+f);<br>DR_A *0.5+0.5	Range: 0 -1
DR_A	(Converted) DR_A_=(n-f)/(n+f):<br>DR_B *2-1	Range: -1 -1
SEM_DR_B	(Converted) SEM corresponding to DR_B:<br>SEM_A / 2	
SEM_DR_A	(Converted) SEM corresponding to DR_A:<br>SEM_B * 2	

A3. DR conversion formula's

A conversion formula was developed to convert the DR that results from one formula into the other. The original two different DR formulas are as follows:

$\mathrm{DR\_A} = \frac{\mathrm{Tnovel} - \mathrm{Tfamiliar}}{\mathrm{Tnovel} + \mathrm{Tfamiliar}}$ or $\mathrm{DR\_B} = \frac{\mathrm{Tnovel}}{\mathrm{Tnovel} + \mathrm{Tfamiliar}}$

DR_A calculates the difference between the exploration time of both objects (T_{novel}-$T_{familiar}$) divided by the total amount of object exploration (T_{total}) during the test phase. DR_A ranges from -1 to 1, making 0 the score at which both objects are explored equally. DR_B calculates the fraction of exploration of the novel object by dividing T_{novel} by T_{total}. DR_B has a range from 0 to 1, this makes 0.5 the score at which animals explore both objects for an equal amount of time.

All DRs that originated from DR_B were converted to scores comparable with DR_A.

To calculate the conversion-factor the formulas were equated to each other. A multiplication factor (*x*) was added to DR_B to correct for the broader range of DR_A (-1 to 1) compared to DR_B (0 to 1). Besides a value (*y*) was added to correct for the shift of the score at which both objects are explored for an equal amount of time (DR_A = 0 and DR_B = 0.5). This results in the following equation:

$$\frac{Tnovel - Tfamiliar}{Tnovel + Tfamiliar} = \frac{Tnovel}{Tnovel + Tfamiliar} x + y$$

To determine the values of *x* and y, two points (T_{novel}, $T_{familiar}$) were inserted in the equation. These points were point 1 (*a,b*) and point 2 (*c,d*). This gives;

1. $(a-b)/(a+b) = a/(a+b)\,x + y$ **and**
2. $(c-d)/(c+d) = c/(c+d)\,x + y \rightarrow y = (c-d)/(c+d) - c/(c+d)\,x$

↓Insert this *y* value into equation 1, this yields;

$$(a-b)/(a+b) = a/(a+b)\,x + (c-d)/(c+d) - c/(c+d)\,x$$
$$(a/(a+b) - c/(c+d))x = (a-b)/(a+b) - (c-d)/(c+d)$$
$$\big(a(c+d) - c(a+b)\big)x = (a-b)(c+d) - (a+b)(c-d)$$
$$(ac+ad-ac-bc)x = ac+ad-bc-bd-ac+ad-bc+bd$$

$(ad-bc)x = 2ad - 2bc$

$x = (2ad-2bc)/(ad-bc)$

$$x = 2$$

↓Insert *x*=2 back into the *y*-equation that resulted from equation 2, this gives;

$$y = (c-d)/(c+d) - c/(c+d) * 2$$
$$y = (c-d-2c)/(c+d)$$
$$y = (-c-d)/(c+d)$$
$$y = -1$$

Now the *x* and *y* values are known they can be inserted in the conversion formula; $\frac{Tnovel-Tfamiliar}{Tnovel+Tfamiliar} = 2 * \frac{Tnovel}{Tnovel+Tfamiliar} - 1$→ This way DR_B scores can be converted by multiplying the original score by 2 and subtracting 1; $DR\_A = DR\_B * 2 - 1$.

Final conversion formula DR: $\mathbf{DR\_A = 2 * DR\_B - 1}$

As the range of DR_A is twice as large as the range of DR_B, the SEM of DR_B is multiplied by 2 to create the SEM of DR_A.

Final conversion formula SEM: SEM DR_A = SEM DR_B * 2

A4. Summary quality scores formulas

Randomization score = Sequence generation (yes = 1, no/unclear = 0) + Baseline characteristics (yes = 1, no/unclear = 0) + Allocation concealment (yes = 1, no/unclear = 0) + Random housing (yes = 1, no/unclear = 0) + Random outcome assessment (yes = 1, no/unclear = 0)

Blinding score = Blinding intervention (yes = 1, no/unclear = 0) + Blinding (analysis) (yes = 1, no/unclear = 0)

Reporting score = Incomplete outcome data (yes = 1, no/unclear = 0) + Selective outcome reporting (yes = 1, no/unclear = 0)

Summary quality score = Randomization score + Blinding score + Reporting score

A5. Summary score formulas

Context difference score (Context.Difference.Score) = Floor (Different = 4, Similar = 0) + Walls (Different = 3, Similar = 0) + Shape (Different = 1, Similar = 0) + Rooms (Different = 1, Similar = 0).

Arousal prior to OIC (Arousal.Prior) = Animal origin (purchased = 1, in-house breeding = 0) + Previous participation in experiments (Yes = 1, No = 0) + Previous participation in Object Recognition tasks (Yes = 0, No = 1) + Type of control group (Sham operation = 2, Sham injection = 1, Undisturbed = 0) + Ad libitum food access (Yes = 0, No = 1) + Reversed Day/night cycle (Yes = 0, No =1) + Housing (Group = 0, Single = 1).

Arousal related to OIC habituation procedures (Arousal.Task.Habituation) = Habituation to experimental boxes (Yes = 0, No = 1) + Habituation to objects (Yes = 0, Partly = 0, No = 1, n.a. = 0) + Habituation with cage mates (Yes = 0, Partly = 0, No = 1, n.a. = 0). Note, n.a. (not applicable) was assigned to studies without habituation phase.

Arousal.Total = Arousal prior to OIC + Arousal related to OIC habituation procedures

A6. Study characteristics

Table A2 Study characteristics of included studies
not included in meta-analysis, only included in systematic review

Study	Species	Sample size
(Ameen-Ali, Eacott, and Easton 2012)	Rats	5
(Asiminas et al. 2019)	Rats	12, 12, 12
(Balderas et al. 2008)	Rats	7, 5, 7, 7, 6, 6, 8, 10
(Barsegyan, McGaugh, and Roozendaal 2014)	Rats	13,12
(Bekinschtein et al. 2013)	Rats	8.5
(Bukhari, Clark, and Williamson 2018)	Rats	5.25, 5.25, 7.25, 7.25
(Chomiak et al. 2014)	Rats	5
(Czerniawski et al. 2015)	Rats	12
(Dix and Aggleton 1999)	Rats	14
(Heimer-McGinn et al. 2017)	Rats	6
(Kanatsou et al. 2015)	Mice	14
(La Spina et al. 2019)	Rats	7, 7
(Langston and Wood 2010)	Rats	13
(Lee et al. 2014) #	Rats	10

(Ji-Tao Li et al. 2013)	Rats	10
(Ji-Tao Li et al. 2011)	Rats	11, 12
(J-T Li et al. 2015)	Rats	8, 9
(Liu et al. 2018)	Rats	11, 11
(Lomeli et al. 2017)	Rats	25
(MacIlvane et al. 2016)	Rats	8, 8
(Martínez et al. 2014) #	Rats	
(Morici et al. 2018)	Mice	12, 13, 13.5, 13.5, 14, 14
(Mumby 2002)	Rats	14
(Mychasiuk et al. 2015)	Rats	3, 3
(Noble et al. 2019)	Rats	11, 12
(Norman and Eacott 2005)	Rats	11, 11
(Pillai et al. 2018)	Mice	6
(Ramsaran, Sanders, and Stanton 2016)	Rats	9, 13, 15, 10, 10, 13, 13, 10, 11
(Ramsaran, Westbrook, and Stanton 2016)	Rats	16, 21, 24, 20
(Sasaki Russell et al. 2019) #	Rats	12
(Sanders, Heroux, and Stanton 2020)	Rats	22.75, 22.75
(Sanderson et al. 2011)	Mice	8, 16
(Schulz et al. 2011)	Rats	9, 9
(Sigwald et al. 2019)	Rats	6
(Spanswick and Dyck 2012)	Mice	5
(Spanswick and Sutherland 2010)	Rats	10
(Tam, Bonardi, and Robinson 2015)	Rats	6, 15, 6, 15
(Vandrey et al. 2020) #	Mice	17
(Wilson et al. 2013)	Rats	7, 8
(Woodward et al. 2018)	Rats	16
(Zhao et al. 2007)	Mice	10.5

References

Ameen-Ali, K.E., M.J. Eacott, and A Easton. 2012. "A New Behavioural Apparatus to Reduce Animal Numbers in Multiple Types of Spontaneous Object Recognition Paradigms in Rats." *Journal of Neuroscience Methods* 211 (1): 66–76. https://doi.org/10.1016/j.jneumeth.2012.08.006.

Asiminas, Antonis, Adam D Jackson, Susana R Louros, Sally M Till, Teresa Spano, Owen Dando, Mark F Bear, et al. 2019. "Sustained Correction of Associative Learning Deficits after Brief, Early Treatment in a Rat Model of Fragile X Syndrome." *Science Translational Medicine* 11 (494). https://doi.org/10.1126/scitranslmed.aao0498.

Balderas, Israela, Carlos J Rodriguez-Ortiz, Paloma Salgado-Tonda, Julio Chavez-Hurtado, James L McGaugh, and Federico Bermudez-Rattoni. 2008. "The Consolidation of Object and Context Recognition Memory Involve Different Regions of the Temporal Lobe." *Learning & Memory (Cold Spring Harbor, N.Y.)* 15 (9): 618–24. https://doi.org/10.1101/lm.1028008.

Barsegyan, Areg, James L McGaugh, and Benno Roozendaal. 2014. "Noradrenergic Activation of the Basolateral Amygdala Modulates the Consolidation of Object-in-Context Recognition Memory." *Frontiers in Behavioral Neuroscience* 8 (May): 160. https://doi.org/10.3389/fnbeh.2014.00160.

Bekinschtein, P., M. C. Renner, M. C. Gonzalez, and N. Weisstaub. 2013. "Role of Medial Prefrontal Cortex Serotonin 2A Receptors in the Control of Retrieval of Recognition Memory in Rats." *Journal of Neuroscience* 33 (40): 15716–25. https://doi.org/10.1523/JNEUROSCI.2087-13.2013.

Bukhari, Syed Hussain F, Olivia E Clark, and Lauren L Williamson. 2018. "Maternal High Fructose Diet and Neonatal Immune Challenge Alter Offspring Anxiety-like Behavior and Inflammation across the Lifespan." *Life Sciences* 197 (March): 114–21. https://doi.org/10.1016/j.lfs.2018.02.010.

Chomiak, T, J Hung, A Cihal, J Dhaliwal, M I Baghdadwala, A Dzwonek, P Podgorny, and B Hu. 2014. "Auditory-Cued Sensorimotor Task Reveals Disengagement Deficits in Rats Exposed to the Autism-Associated Teratogen Valproic Acid." *Neuroscience* 268 (May): 212–20. https://doi.org/10.1016/j.neuroscience.2014.02.049.

Czerniawski, Jennifer, Teiko Miyashita, Gail Lewandowski, and John F Guzowski. 2015. "Systemic Lipopolysaccharide Administration Impairs Retrieval of Context-Object Discrimination, but Not Spatial, Memory: Evidence for Selective Disruption of Specific Hippocampus-Dependent Memory Functions during Acute Neuroinflammation." *Brain, Behavior, and Immunity* 44 (February): 159–66. https://doi.org/10.1016/j.bbi.2014.09.014.

Dix, S L, and J P Aggleton. 1999. "Extending the Spontaneous Preference Test of Recognition: Evidence of Object-Location and Object-Context Recognition." *Behavioural Brain Research* 99 (2): 191–200. https://doi.org/10.1016/s0166-4328(98)00079-5.

Heimer-McGinn, Victoria R, Devon L Poeta, Krishan Aghi, Methma Udawatta, and Rebecca D Burwell. 2017. "Disconnection of the Perirhinal and Postrhinal Cortices Impairs Recognition of Objects in Context But Not Contextual Fear Conditioning." *The Journal of Neuroscience : The Official Journal of the Society for Neuroscience* 37 (18): 4819–29. https://doi.org/10.1523/JNEUROSCI.0254-17.2017.

Kanatsou, Sofia, Laura E Kuil, Marit Arp, Melly S Oitzl, Anjanette P Harris, Jonathan R Seckl, Harm J Krugers, and Marian Joels. 2015. "Overexpression of Mineralocorticoid Receptors Does Not Affect Memory and Anxiety-like Behavior in Female Mice." *Frontiers in Behavioral Neuroscience* 9 (July): 182. https://doi.org/10.3389/fnbeh.2015.00182.

Langston, Rosamund F, and Emma R Wood. 2010. "Associative Recognition and the Hippocampus: Differential Effects of Hippocampal Lesions on Object-Place, Object-Context and Object-Place-Context Memory." *Hippocampus* 20 (10): 1139–53. https://doi.org/10.1002/hipo.20714.

Lee, Bradley H, John Thomas Chan, Obhi Hazarika, Laszlo Vutskits, and Jeffrey W Sall. 2014. "Early Exposure to Volatile Anesthetics Impairs Long-Term Associative Learning and Recognition Memory." *PloS One* 9 (8): e105340. https://doi.org/10.1371/journal.pone.0105340.

Li, J-T, Y-Y Zhao, H-L Wang, X-D Wang, Y-A Su, and T-M Si. 2015. "Long-Term Effects of Neonatal Exposure to MK-801 on Recognition Memory and Excitatory–Inhibitory Balance in Rat Hippocampus." *Neuroscience* 308 (November): 134–43. https://doi.org/10.1016/j.neuroscience.2015.09.003.

Li, Ji-Tao, Yu Feng, Yun-Ai Su, Xiao-Dong Wang, and Tian-Mei Si. 2013. "Enhanced Interaction among ErbB4, PSD-95 and NMDAR by Chronic MK-801 Treatment Is Associated with Behavioral Abnormalities." *Pharmacology Biochemistry and Behavior* 108 (July): 44–53. https://doi.org/10.1016/j.pbb.2013.04.008.

Li, Ji-Tao, Yun-Ai Su, Chun-Mei Guo, Yu Feng, Yang Yang, Run-Hu Huang, and Tian-Mei Si. 2011. "Persisting Cognitive Deficits Induced by Low-Dose, Subchronic Treatment with MK-801 in Adolescent Rats." *European Journal of Pharmacology* 652 (1–3): 65–72. https://doi.org/10.1016/j.ejphar.2010.10.074.

Liu, Xiao, Jitao Li, Chunmei Guo, Hongli Wang, Yaxin Sun, Han Wang, Yun-Ai Su, Keqing Li, and Tianmei Si. 2018. "Olanzapine Reverses MK-801-Induced Cognitive Deficits and Region-Specific Alterations of NMDA Receptor Subunits." *Frontiers in Behavioral Neuroscience* 11 (January): 260. https://doi.org/10.3389/fnbeh.2017.00260.

Lomeli, Naomi, Kaijun Di, Jennifer Czerniawski, John F Guzowski, and Daniela A Bota. 2017. "Cisplatin-Induced Mitochondrial Dysfunction Is Associated with Impaired Cognitive Function in Rats." *Free Radical Biology and Medicine* 102 (January): 274–86. https://doi.org/10.1016/j.freeradbiomed.2016.11.046.

MacIlvane, Nicole M, Joseph M Pochiro, Nicole R Hurwitz, Molly J Goodfellow, and Derick H Lindquist. 2016. "Recognition Memory Is Selectively Impaired in Adult Rats Exposed to Binge-like Doses of Ethanol during Early Postnatal Life." *Alcohol* 57 (December): 55–63. https://doi.org/10.1016/j.alcohol.2016.09.027.

Martínez, María Cecilia, María Eugenia Villar, Fabricio Ballarini, and Haydée Viola. 2014. "Retroactive Interference of Object-in-Context Long-Term Memory: Role of Dorsal Hippocampus and Medial Prefrontal Cortex." *Hippocampus* 24 (12): 1482–92. https://doi.org/10.1002/hipo.22328.

Morici, Juan Facundo, Magdalena Miranda, Francisco Tomás Gallo, Belén Zanoni, Pedro Bekinschtein, and Noelia V Weisstaub. 2018. "5-HT2a Receptor in MPFC Influences Context-Guided Reconsolidation of Object Memory in Perirhinal Cortex." *ELife* 7 (May). https://doi.org/10.7554/eLife.33746.

Mumby, Dave G. 2002. "Hippocampal Damage and Exploratory Preferences in Rats: Memory for Objects, Places, and Contexts." *Learning & Memory* 9 (2): 49–57. https://doi.org/10.1101/lm.41302.

Mychasiuk, Richelle, Harleen Hehar, Linda van Waes, and Michael J Esser. 2015. "Diet, Age, and Prior Injury Status Differentially Alter Behavioral Outcomes Following Concussion in Rats." *Neurobiology of Disease* 73 (January): 1–11. https://doi.org/10.1016/j.nbd.2014.09.003.

Noble, Emily E, Ted M Hsu, Joanna Liang, and Scott E Kanoski. 2019. "Early-Life Sugar Consumption Has Long-Term Negative Effects on Memory Function in Male Rats." *Nutritional Neuroscience* 22 (4): 273–83. https://doi.org/10.1080/1028415X.2017.1378851.

Norman, G, and M J Eacott. 2005. "Dissociable Effects of Lesions to the Perirhinal Cortex and the Postrhinal Cortex on Memory for Context and Objects in Rats." *Behavioral Neuroscience* 119 (2): 557–66. https://doi.org/10.1037/0735-7044.119.2.557.

Pillai, Anup G, Marit Arp, Els Velzing, Sylvie L Lesuis, Mathias V Schmidt, Florian Holsboer, Marian Joëls, and Harm J Krugers. 2018. "Early Life Stress Determines the Effects of Glucocorticoids and Stress on

Hippocampal Function: Electrophysiological and Behavioral Evidence Respectively." *Neuropharmacology* 133 (May): 307–18. https://doi.org/10.1016/j.neuropharm.2018.02.001.

Ramsaran, Adam I, Hollie R Sanders, and Mark E Stanton. 2016. "Determinants of Object-in-Context and Object-Place-Context Recognition in the Developing Rat." *Developmental Psychobiology* 58 (7): 883–95. https://doi.org/10.1002/dev.21432.

Ramsaran, Adam I, Sara R Westbrook, and Mark E Stanton. 2016. "Ontogeny of Object-in-Context Recognition in the Rat." *Behavioural Brain Research* 298 (Pt A): 37–47. https://doi.org/10.1016/j.bbr.2015.04.011.

Sanders, Hollie R, Nicholas A Heroux, and Mark E Stanton. 2020. "Infant Rats Can Acquire, but Not Retain Contextual Associations in Object-in-context and Contextual Fear Conditioning Paradigms." *Developmental Psychobiology* 62 (8): 1158–64. https://doi.org/10.1002/dev.21980.

Sanderson, David J, Emma Hindley, Emily Smeaton, Nick Denny, Amy Taylor, Chris Barkus, Rolf Sprengel, Peter H Seeburg, and David M Bannerman. 2011. "Deletion of the GluA1 AMPA Receptor Subunit Impairs Recency-Dependent Object Recognition Memory." *Learning & Memory* 18 (3): 181–90. https://doi.org/10.1101/lm.2083411.

Sasaki Russell, Jennifer M, Gregory A Chinn, Deenu Maharjan, Yasmine Eichbaum, and Jeffrey W Sall. 2019. "Female Rats Are More Vulnerable to Lasting Cognitive Impairment after Isoflurane Exposure on Postnatal Day 4 than 7." *British Journal of Anaesthesia* 122 (4): 490–99. https://doi.org/10.1016/j.bja.2018.12.008.

Schulz, Kalynn M, Jennifer N Pearson, Eric W Neeley, Ralph Berger, Sherry Leonard, Catherine E Adams, and Karen E Stevens. 2011. "Maternal Stress during Pregnancy Causes Sex-Specific Alterations in Offspring Memory Performance, Social Interactions, Indices of Anxiety, and Body Mass." *Physiology & Behavior* 104 (2): 340–47. https://doi.org/10.1016/j.physbeh.2011.02.021.

Sigwald, Eric L, Elena A Bignante, Soledad de Olmos, and Alfredo Lorenzo. 2019. "Fear-Context Association during Memory Retrieval Requires Input from Granular to Dysgranular Retrosplenial Cortex." *Neurobiology of Learning and Memory* 163 (September): 107036. https://doi.org/10.1016/j.nlm.2019.107036.

Spanswick, Simon C., and Robert J. Sutherland. 2010. "Object/Context-Specific Memory Deficits Associated with Loss of Hippocampal Granule Cells after Adrenalectomy in Rats." *Learning & Memory* 17 (5): 241–45. https://doi.org/10.1101/lm.1746710.

Spanswick, Simon C, and Richard H Dyck. 2012. "Object/Context Specific Memory Deficits Following Medial Frontal Cortex Damage in Mice." Edited by Andrew Iwaniuk. *PLoS ONE* 7 (8): e43698. https://doi.org/10.1371/journal.pone.0043698.

Spina, Martina La, Gabriele Sansevero, Lucia Biasutto, Mario Zoratti, Roberta Peruzzo, Nicoletta Berardi, Alessandro Sale, and Michele Azzolini. 2019. "Pterostilbene Improves Cognitive Performance in Aged Rats: An in Vivo Study." *Cellular Physiology and Biochemistry : International Journal of Experimental Cellular Physiology, Biochemistry, and Pharmacology* 52 (2): 232–39. https://doi.org/10.33594/000000017.

Tam, Shu K.E., Charlotte Bonardi, and Jasper Robinson. 2015. "Relative Recency Influences Object-in-Context Memory." *Behavioural Brain Research* 281 (March): 250–57. https://doi.org/10.1016/j.bbr.2014.12.024.

Vandrey, Brianna, Derek L.F. Garden, Veronika Ambrozova, Christina McClure, Matthew F Nolan, and James A Ainge. 2020. "Fan Cells in Layer 2 of the Lateral Entorhinal Cortex Are Critical for Episodic-like Memory." *Current Biology* 30 (1): 169-175.e5. https://doi.org/10.1016/j.cub.2019.11.027.

Wilson, David I.G., Rosamund F. Langston, Magdalene I. Schlesiger, Monica Wagner, Sakurako Watanabe, and James A. Ainge. 2013. "Lateral Entorhinal Cortex Is Critical for Novel Object-context Recognition." *Hippocampus* 23 (5): 352–66. https://doi.org/10.1002/hipo.22095.

Woodward, N C, A Haghani, R G Johnson, T M Hsu, A Saffari, C Sioutas, S E Kanoski, C E Finch, and T E Morgan. 2018. "Prenatal and Early Life Exposure to Air Pollution Induced Hippocampal Vascular Leakage and Impaired Neurogenesis in Association with Behavioral Deficits." *Translational Psychiatry* 8 (1): 261. https://doi.org/10.1038/s41398-018-0317-1.

Zhao, Qi, Yukihisa Murakami, Michihisa Tohda, Ryosuke Obi, Yutaka Shimada, and Kinzo Matsumoto. 2007. "Chotosan, a Kampo Formula, Ameliorates Chronic Cerebral Hypoperfusion-Induced Deficits in Object Recognition Behaviors and Central Cholinergic Systems in Mice." *Journal of Pharmacological Sciences* 103 (4): 360–73. https://doi.org/10.1254/jphs.FP0061457.

A7. Systematic review table

Table A3. Systematic overview of Variation in the OIC	
**Characteristic**	**Observations (N = 97[1])**
**Sample size**	3.0 - 25.0; 10.8; 10.9 (4.6)
Not reported	13
**Species**	
Mice	8 / 97 (8.2%)
Rat	89 / 97 (92%)
**Strain**	
C57BL/6	3 / 94 (3.2%)
Dark Agouti rats	2 / 94 (2.1%)
ICR	1 / 94 (1.1%)
Lister hooded rats	8 / 94 (8.5%)
Long-Evans rats	22 / 94 (23%)
pigmented DA strain	1 / 94 (1.1%)
Sprague-Dawley	26 / 94 (28%)
Sv/Ev mice	1 / 94 (1.1%)
Tg(Sim1cre)KH21Gsat/Mmucd	1 / 94 (1.1%)
Wistar rats	29 / 94 (31%)
Not reported	3
**Sex**	
Female	6 / 95 (6.3%)
Male	67 / 95 (71%)
Mixed	22 / 95 (23%)
Not reported	2
**Origin**	
In-house breeding	46 / 74 (62%)
Supplier	28 / 74 (38%)
Not reported	23
**Age**	
Adolescent	11 / 90 (12%)
Adult	67 / 90 (74%)
Juvenile	10 / 90 (11%)
Senior	2 / 90 (2.2%)
Not reported	7
**Type.Control**	
Undisturbed	36 / 97 (37%)
Sham injection	38 / 97 (39%)
Sham surgery	23 / 97 (24%)

Table A3. Systematic overview of Variation in the OIC	
**Characteristic**	**Observations (N = 97[1])**
**Housing**	
Group	67 / 92 (73%)
Single	25 / 92 (27%)
Not reported	5
**Reversed day/night cycle**	
No	78 / 80 (98%)
Yes	2 / 80 (2.5%)
Not reported	17
**Previous experiment**	
No	79 / 95 (83%)
Yes	16 / 95 (17%)
Not reported	2
**Previous object recognition task**	
No	61 / 95 (64%)
Yes	34 / 95 (36%)
Not reported	2
**Ad libitum food & water**	
No	3 / 93 (3.2%)
Yes	90 / 93 (97%)
Not reported	4
**Handling [#]**	
Yes	52 / 52 (100%)
Not reported	45
**Context.Floor**	
Different	68 / 80 (85%)
Similar	12 / 80 (15%)
Not reported	17
**Context.Wall**	
Different	88 / 95 (93%)
Similar	7 / 95 (7.4%)
Not reported	2
**Context.Shape**	
Different	37 / 96 (39%)
Similar	59 / 96 (61%)
Not reported	1
**Context.Room**	
Different	25 / 56 (45%)
Similar	31 / 56 (55%)

**Table A3. Systematic overview of Variation in the OIC**	
**Characteristic**	**Observations (N = 97[1])**
Not reported	41
**Context.Size.A**	360 - 10,000; 2,400; 3,051 (1,823)
Not reported	3
**Context.Size.B**	289 - 10,000; 2,400; 3,101 (1,835)
Not reported	3
**Object.Material**	
Different	60 / 78 (77%)
Similar	18 / 78 (23%)
Not reported	19
**Object.Size** #	
Different	33 / 62 (53%)
Similar	29 / 62 (47%)
Not reported	35
**Context.Order**	
AA	32 / 95 (34%)
AB	23 / 95 (24%)
Both	40 / 95 (42%)
Not reported	2
**Transport.Habituation** #	
Yes	18 / 18 (100%)
Not reported	79
**Testroom.Habituation** #	
Yes	22 / 22 (100%)
Not reported	75
**Habituation.with.Cagemates**	
n.a.	19 / 72 (26%)
No	43 / 72 (60%)
Partly	8 / 72 (11%)
Yes	2 / 72 (2.8%)
Not reported	25
**Context.Habituation.Freq**	0.00 - 8.00; 3.00; 2.91 (1.89)
Not reported	8
**Habituation Time Trial (min)**	0 - 20; 10; 9 (6)
Not reported	17
**Habituation Time Total Context (min)**	0 - 120; 30; 27 (19)
Not reported	9
**Object.Habituation**	
n.a.	7 / 88 (8.0%)

**Table A3. Systematic overview of Variation in the OIC**	
**Characteristic**	**Observations (N = 97[1])**
No	67 / 88 (76%)
Partly	10 / 88 (11%)
Yes	4 / 88 (4.5%)
Not reported	9
**Learning Time Trial (min)**	1.8 - 20.0; 5.0; 7.2 (4.4)
**Learning-Learning Delay (min)**	0 - 1,440; 5; 232 (493)
Not reported	4
**Learning.Order.Counterbalanced**	
No	3 / 83 (3.6%)
Yes	80 / 83 (96%)
Not reported	14
**Learning Exploration Time A (sec)** #	15 - 158; 56; 59 (38)
Not reported	56
**Learning Exploration Time B (sec)** #	13 - 160; 56; 56 (39)
Not reported	56
**Learning Exploration Total (sec)** #	27 - 312; 111; 113 (75)
Not reported	54
**Learning-Memory Delay (min)**	1 - 5,760; 120; 743 (876)
Not reported	2
**Memory Time Trial (min)**	0.50 - 10.00; 3.00; 3.26 (1.54)
Not reported	3
**Memory Exploration Time A (sec)** #	24 - 55; 37; 38 (15)
Not reported	93
**Memory Exploration Time B (sec)** #	19 - 58; 45; 42 (18)
Not reported	93
**Memory Exploration Time Familiar (sec)** #	8 - 37; 13; 16 (9)
Not reported	87
**Memory Exploration Time New (sec)** #	11 - 51; 22; 25 (13)
Not reported	87
**Memory Exploration Time Total (sec)** #	11 - 69; 25; 32 (17)
Not reported	64
**Smell Control OIC**	
Yes	75 / 75 (100%)
Not reported	22
**Reuse Bedding OIC** #	
n.a.	43 / 55 (78%)
No	1 / 55 (1.8%)
Partly	1 / 55 (1.8%)

Table A3. Systematic overview of Variation in the OIC

**Characteristic**	**Observations (N = 97[1])**
Yes	10 / 55 (18%)
Not reported	42
**Scoring**	
Both	2 / 73 (2.7%)
Digital	11 / 73 (15%)
Manual	60 / 73 (82%)
Not reported	24
**Observers [#]**	
>1	23 / 54 (43%)
1	20 / 54 (37%)
n.a.	11 / 54 (20%)
Not reported	43
**Scoring Program**	
AnyMaze software	8 / 71 (11%)
Ethovision XT (Noldus)	2 / 71 (2.8%)
Limelight2 program (Actimetrics; Coubourn Instruments)	1 / 71 (1.4%)
Med-PCIV software	1 / 71 (1.4%)
n.a.	57 / 71 (80%)
Topscan	2 / 71 (2.8%)
Not reported	26
**DR.Formula**	
(tnew-tfamiliar)/(tnew+tfamiliar)	36 / 97 (37%)
(tnew)/(tnew+tfamiliar)	39 / 97 (40%)
[(tnew-tfamiliar)/(tnew+tfamiliar)]*100	3 / 97 (3.1%)
[(tnew)/(tnew+tfamiliar)]*100	19 / 97 (20%)

[1]Range; Median; Mean (SD); n / N (%); [#] Variables with more than 1/3 missing values that were not included in the random forest analysis.

A8. Study quality

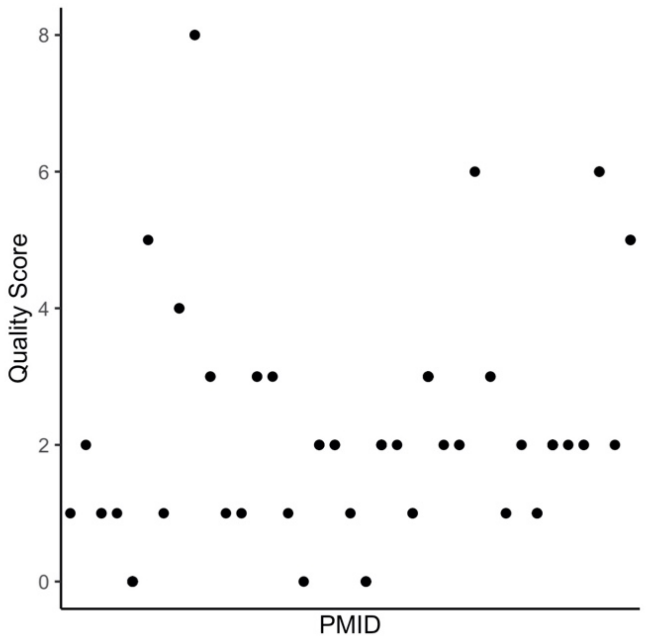

Fig A1. Cumulative study quality based on SYRCLE's risk of bias assessment per article.

A9. Random forest model convergence plot

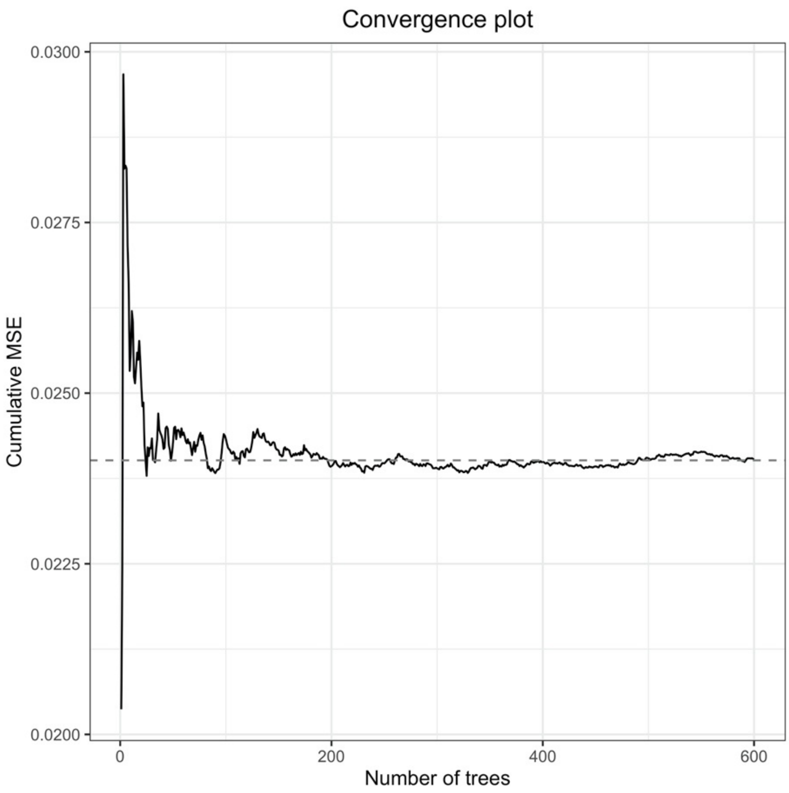

Fig A2. Random forest model convergence.
The stable cumulative MSE indicates good model convergence at 600 trees.

A10. PRISMA 2009 Checklist

From: Moher D, Liberati A, Tetzlaff J, Altman DG, The PRISMA Group (2009). Preferred Reporting Items for Systematic Reviews and Meta-Analyses: The PRISMA Statement. PLoS Med 6(7): e1000097. doi:10.1371/journal.pmed1000097

Section/topic	#	Checklist item	Reported on page #
**TITLE**			
Title	1	Identify the report as a systematic review, meta-analysis, or both.	1
**ABSTRACT**			
Structured summary	2	Provide a structured summary including, as applicable: background; objectives; data sources; study eligibility criteria, participants, and interventions; study appraisal and synthesis methods; results; limitations; conclusions and implications of key findings; systematic review registration number.	2
**INTRODUCTION**			
Rationale	3	Describe the rationale for the review in the context of what is already known.	3-5
Objectives	4	Provide an explicit statement of questions being addressed with reference to participants, interventions, comparisons, outcomes, and study design (PICOS).	5
**METHODS**			
Protocol and registration	5	Indicate if a review protocol exists, if and where it can be accessed (e.g., Web address), and, if available, provide registration information including registration number.	5
Eligibility criteria	6	Specify study characteristics (e.g., PICOS, length of follow-up) and report characteristics (e.g., years considered, language, publication status) used as criteria for eligibility, giving rationale.	6
Information sources	7	Describe all information sources (e.g., databases with dates of coverage, contact with study authors to identify additional studies) in the search and date last searched.	5
Search	8	Present full electronic search strategy for at least one database, including any limits used, such that it could be repeated.	5; Appendix A1
Study selection	9	State the process for selecting studies (i.e., screening, eligibility, included in systematic review, and, if applicable, included in the meta-analysis).	6
Data collection process	10	Describe method of data extraction from reports (e.g., piloted forms, independently, in duplicate) and any processes for obtaining and confirming data from investigators.	6-7
Data items	11	List and define all variables for which data were sought (e.g., PICOS, funding sources) and any assumptions and simplifications made.	Appendix A2
Risk of bias in individual studies	12	Describe methods used for assessing risk of bias of individual studies (including specification of whether this was done at the study or outcome level), and how this information is to be used in any data synthesis.	7-8
Summary measures	13	State the principal summary measures (e.g., risk ratio, difference in means).	7
Synthesis of results	14	Describe the methods of handling data and combining results of studies, if done, including measures of consistency (e.g., I^2) for each meta-analysis.	7-8

Section/topic	#	Checklist item	Reported on page #
Risk of bias across studies	15	Specify any assessment of risk of bias that may affect the cumulative evidence (e.g., publication bias, selective reporting within studies).	7-8
Additional analyses	16	Describe methods of additional analyses (e.g., sensitivity or subgroup analyses, meta-regression), if done, indicating which were pre-specified.	8-9
**RESULTS**			
Study selection	17	Give numbers of studies screened, assessed for eligibility, and included in the review, with reasons for exclusions at each stage, ideally with a flow diagram.	9
Study characteristics	18	For each study, present characteristics for which data were extracted (e.g., study size, PICOS, follow-up period) and provide the citations.	Appendix A6
Risk of bias within studies	19	Present data on risk of bias of each study and, if available, any outcome level assessment (see item 12).	Fig 3
Results of individual studies	20	For all outcomes considered (benefits or harms), present, for each study: (a) simple summary data for each intervention group (b) effect estimates and confidence intervals, ideally with a forest plot.	Fig 4
Synthesis of results	21	Present results of each meta-analysis done, including confidence intervals and measures of consistency.	10
Risk of bias across studies	22	Present results of any assessment of risk of bias across studies (see Item 15).	11; Fig 5; Appendix A8
Additional analysis	23	Give results of additional analyses, if done (e.g., sensitivity or subgroup analyses, meta-regression [see Item 16]).	11-12
**DISCUSSION**			
Summary of evidence	24	Summarize the main findings including the strength of evidence for each main outcome; consider their relevance to key groups (e.g., healthcare providers, users, and policy makers).	14-19
Limitations	25	Discuss limitations at study and outcome level (e.g., risk of bias), and at review-level (e.g., incomplete retrieval of identified research, reporting bias).	15-16
Conclusions	26	Provide a general interpretation of the results in the context of other evidence, and implications for future research.	19-21
**FUNDING**			
Funding	27	Describe sources of funding for the systematic review and other support (e.g., supply of data); role of funders for the systematic review.	22
